# Ovarian stimulation with luteinizing hormone supplementation: the impact of timing on ovarian response and ICSI outcomes

**DOI:** 10.5935/1518-0557.20220022

**Published:** 2023

**Authors:** Amanda Setti, Daniela Braga, Assumpto Iaconelli Jr., Edson Borges Jr.

**Affiliations:** 1 Instituto Sapientiae - Centro de Estudos e Pesquisa em Reprodução Humana Assistida; 2 Fertility Medical Group

**Keywords:** Follicle-stimulating hormone, ICSI, implantation, luteinizing hormone, ovarian stimulation

## Abstract

**Objective:**

To investigate whether the timing of rLH addition to rFSH during controlled ovarian stimulation (COS) impacts ovarian response and the outcomes of intracytoplasmic sperm injection (ICSI) cycles.

**Methods:**

Data of 1278 patients undergoing ICSI between 2015 and 2018, in a private university-affiliated IVF center were analyzed. Patients were divided into groups according to the timing of LH addition to the COS protocol: Group LH-start (n=323), in which LH was administered since day 1 of ovarian stimulation; and Group LH-mid (n=955), in which LH was administered concomitantly with gonadotropin releasing hormone (GnRH) antagonist. Data were also stratified according to female age and response to COS. The outcomes of COS and ICSI were compared between the groups.

**Results:**

For the general group and in patients aged ≥ 35 years, higher blastocyst development rates were in Group LH-mid compared to Group LH-start. In patients with poor response to COS (POR), higher fertilization rate, blastocyst development rate and implantation rate were observed in Group LH-mid.

**Conclusions:**

rLH supplementation in POR patients may improve laboratorial and clinical outcomes when started in the mid-follicular phase, in GnRH antagonist ICSI cycles.

## Introduction

Recombinant follicle-stimulating hormone (rFSH) and luteinizing hormone (rLH), individually or in combination, are the most routinely used gonadotropins for controlled ovarian stimulation (COS) in assisted reproductive technology (ART). Despite their divergent roles, these hormones complement each other during follicle regulation, from recruitment to oocyte ovulation (Raju *et al*., 2013).

One particular onus of the ovarian stimulation is the diminution in releases of endogenous FSH and LH (Mochtar *et al*., 2007), via pituitary blockage with GnRH antagonists. Despite, stimulation with exogenous FSH generally supports follicular development, more than 10% of patients undergoing ART do not respond to FSH stimulation, probably due to lack of LH (Hill *et al*., 2012a). For these patients, there is a strong body of evidence showing that LH supplementation is beneficial (Humaidan *et al*., 2004; Alviggi *et al*., 2011; 2012; Bosch *et al*., 2011; Matorras *et al.*, 2011; Wong *et al*., 2011; Hill *et al*., 2012a; 2012b).

Initially, rLH was commercialized to supplement FSH administration in patients with hypogonadotropic hypogonadism. Later, novel drugs were developed in a fixed combination of 2:1 (150 IU of rFSH and 75 IU of rLH) for the purpose of stimulating follicular development. Then, the use of LH supplementation was recommended to patients with previous poor response (POR) to COS, inadequate ovarian response in the current treatment, and those aged ≥35 years (Wong *et al*., 2011). A meta-analysis of 36 randomized controlled trials and 8125 women undergoing ART investigating the effectiveness of rLH addition to rFSH for COS compared to rFSH alone showed that the use of rLH combined with rFSH may lead to more ongoing pregnancies than rFSH alone (Mochtar *et al*., 2017).

To the best of our knowledge, there is limited evidence that the timing of rLH addition to rFSH during COS may impact the ovarian response or the outcomes of ICSI. Only one study, with a limited casuistic, demonstrated improved ovarian response, embryo quality and pregnancy rate with LH supplementation from GnRH antagonist administration day, in estimated POR patients. On the other hand, improved endometrial thickness was observed by administering LH from the start of FSH administration (Gizzo *et al*., 2015). The objective of the present study was to further investigate this hypothesis in a larger population, and in subpopulations of patients stratified by age and response to COS.

## Materials and Methods

### Experimental design, patients, and inclusion and exclusion criteria

This historical cohort study included data obtained via chart review of 1278 ICSI cycles performed in 1278 patients between 2015 and 2018, in a private university-affiliated IVF center. In the first analysis, patients were divided into two groups according to the timing of LH addition to the COS protocol: Group LH-start (n=323), in which LH was administered since day 1 of ovarian stimulation; and Group LH-mid (n=955), in which LH was administered from the sixth day of stimulus on. Then, data were stratified according to female age (< 35 years-old, n=283, and ≥ 35 years-old, n=995) and response to COS (POR: ≤ 4 retrieved oocytes, n= 423, and normal response: > 5 retrieved oocytes, n= 855). The response to COS and ICSI outcomes were compared among the groups.

The inclusion criteria were as follows: couples with primary infertility undergoing their first or second ICSI cycle, which had fresh embryo transfer on day 5 of embryo development.

The exclusion criteria were as follows: Female patients undergoing ICSI cycles with vitrified/thawed or donated oocytes, surgical sperm retrieval, cryopreserved sperm, and vitrified/thawed embryo transfer.

Ovarian response to COS and ICSI outcomes were compared between the groups.

All patients signed a written informed consent form. The study was approved by the local Institutional Review Board.

### Controlled ovarian stimulation

For patients in the LH-start group, ovarian stimulation was started on day 3 of cycle with a fixed ratio combination of rFSH 300 IU and rLH 150 IU (Pergoveris^®^, Merck Serono S.p.A, Bari, Italy) until the day of hCG trigger.

For patients in the LH-mid group, ovarian stimulation was also started on day 3 of cycle, but with administration of rFSH 300 IU monotherapy (Gonal-f, Serono, Geneva, Switzerland) for five days. The gonadotropin dose was adjusted with the introduction of rLH (Pergoveris), which was started concomitantly with GnRH antagonist, and maintained until the day of hCG trigger.

The following steps were the same for both groups. When at least two follicles ≥14 mm were visualized, pituitary blockage was performed using gonadotropin-releasing hormone (GnRH) antagonist (Cetrotide^®^; Merck KGaA, Darmstadt, Germany). When three or more follicles attained a mean diameter of ≥ 17mm and adequate serum estradiol levels were observed, final follicular maturation was triggered by the administration of r-hCG (250 µg, Ovidrel^®^, Merck KGaA, Geneva, Switzerland) or GnRH agonist (triptorelin 0.2 mg, Gonapeptyl; Ferring GmbH, Kiel, Germany or leuprolide acetate 2.0mg, Lupron Kit™, Abbott S.A Societé Française des Laboratoires, Paris, France). Oocyte retrieval was performed 35 hours later.

### Oocyte preparation

Retrieved oocytes were cultivated in culture media drops (Global for fertilization, LifeGlobal, Guilford, USA) covered with paraffin oil (Paraffin oil P.G., LifeGlobal) for 4 h before cumulus cell denudation. The surrounding cumulus cells were removed enzymatically by exposure to a HEPES-buffered medium containing hyaluronidase (80 IU/ml, LifeGlobal), and then mechanically by gently pipetting with a hand-drawn Pasteur pipette (Humagen Fertility Diagnostics, Charlottesville, USA). Oocytes were incubated for 1h before ICSI.

Oocyte maturity was assessed using an inverted Nikon Diaphot microscope with a Hoffmann modulation contrast system under 400x magnification (Eclipse TE 300 microscope, Nikon, Tokyo, Japan), just before sperm injection (4 hours after retrieval). Oocytes that had released the first polar body (MII stage) were used for ICSI.

### Intracytoplasmic sperm injection

Intracytoplasmic sperm injection was performed according to Palermo *et al*. (1992). Sperm selection was analyzed at 400x magnification using an inverted Nikon Eclipse TE 300 microscope. The injection was performed in a micro-injection dish prepared with buffered medium (Global w/HEPES, LifeGlobal, Guilford, USA) covered with paraffin oil, on an inverted microscope heated stage (37.0°C ± 0.5°C). Fertilization was confirmed by the presence of two pronuclei (PN) and the extrusion of the second polar body 16 - 18h post-ICSI. Embryos were cultured in 50-µL drops of culture medium (Global®, LifeGlobal, Guilford, USA) covered with paraffin oil, in a humidified atmosphere under 6% CO_2_, at 37°C, for five days.

### Embryo quality and embryo transfer

Embryos were morphologically evaluated on days three and five of development using an inverted Nikon Diaphot microscope with a Hoffmann modulation contrast system (Eclipse TE 300 microscope, Nikon, Tokyo, Japan) under 400x magnification.

The blastocyst development rate was defined as the number of embryos that reached blastocyst stage at day five by the number of fertilized oocytes in each cycle. On day 5, one to two embryos were transferred per patient, depending on maternal age and embryo quality, using a soft catheter with transabdominal ultrasound guidance.

### Clinical follow-up

A serum pregnancy test was performed 10 days after embryo transfer. Women with a positive βhCG test underwent a transvaginal ultrasound scan after two weeks. Clinical pregnancy was confirmed when at least one intrauterine gestational sac with fetal heartbeat was detected. Implantation rate was calculated as the number of gestational sacs divided by the number of embryos transferred. Clinical pregnancy rates were calculated per embryo transfer. Miscarriage was defined as pregnancy loss before 20 weeks gestation.

### Data analysis and statistics

Post hoc power analysis was calculated, given α of 5%, sample size of 1278, and effect size for implantation rate. The achieved power was superior to 99%. The calculation was performed using G*Power 3.1.7.

In the first analysis, demographics (female and male ages, and female BMI), response to COS (FSH dose, estradiol level on trigger day, number of follicles and retrieved oocytes, oocyte yield, and MII oocyte rates), and the outcomes of ICSI (fertilization rate, blastocyst development rate, cycles with embryo transfer rate, implantation rate, pregnancy rate and miscarriage rate) were compared between the groups using generalized linear models followed by Bonferroni post hoc test. Then, data were stratified according to female age and response to COS and were re-analyzed as above-mentioned.

Data are expressed as mean ± standard error for continuous variables or percentages for dichotomous variables, and p-values. *p*-value was significant at 5% level (<0.05). The analysis was performed using SPSS Statistics 21 (IBM, New York, New York, USA).

## Results

For the general group, higher blastocyst development rate was observed in Group LH-mid compared to Group LH-start (40.8 ± 32.6 vs. 33.0 ± 31.9, *p*=0.012) (Table 1).

**Table 1:** Descriptive analysis of patients’ demographics, response to COS and laboratorial ICSI outcomes

Variable	Group LH-start (n=323)	Group LH-mid (n=955)	*p*-value
Female age	37.46 ± 4.38	37.48 ± 4.16	0.957
Male age	39.69 ± 6.59	39.52 ± 6.32	0.711
BMI	24.70 ± 3.93	24.44 ± 4.00	0.331
FSH dose (IU)	2474.66 ± 813.70	2567.03 ± 659.76	0.066
LH dose (IU)	1236.69 ± 406.75	980.84 ± 482.16	<0.001
Estradiol level (pg/mL)	2009.12 ± 1978.21	1981.58 ± 1884.36	0.890
Follicles (n)	11.56 ± 9.66	12.66 ± 9.71	0.079
Retrieved oocytes (n)	8.32 ± 7.53	9.29 ± 7.86	0.052
Oocyte yield (%)	70.12 ± 24.40	70.83 ± 24.09	0.651
MII oocyte rate (%)	73.37 ± 22.93	72.18 ± 23.82	0.443
Fertilization rate (%)	76.93 ± 23.33	73.91 ± 25.66	0.084
Normal cleavage speed rate (%)	74.10 ± 29.15	73.17 ± 28.77	0.648
D3 high -quality embryos rate (%)	43.93 ± 25.88	40.55 ± 24.59	0.293
Blastocyst development rate (%)	33.00 ± 31.95	40.81 ± 32.62	0.012
Frozen embryos (n)	4.36 ± 4.82	4.21 ± 4.05	0.761
Endometrial thickness (mm)	10.49 ± 2.20	10.59 ± 2.41	0.647
Embryos transferred (n)	1.33 ± 0.59	1.32 ± 0.54	0.877
Cycles with embryo transfer (%)	56.04 (181/323)	54.76 (523/955)	0.698
Implantation rate (%)	24.2 ± 40.5	26.1 ± 40.9	0.593
Pregnancy rate (%)	28.7 (52/181)	31.5 (165/523)	0.514
Miscarriage rate (%)	11.5 (6/52)	13.3 (22/165)	0.817

Note: values are mean ± standard deviation, unless otherwise noted. ICSI - intracytoplasmic sperm injection, COS - controlled ovarian stimulation, rFSH - recombinant follicle stimulating hormone, rLH - recombinant luteinizing hormone, BMI - body mass index, IU - international unit, MII - metaphase II, D3 - day 3 of embryo development.

In patients aged < 35 years, no significant differences were observed between the groups (Table 2).

**Table 2:** Descriptive analysis of patients’ demographics, response to COS and laboratorial ICSI outcomes in patients aged < 35 years

Variable	Group LH-start (n=70)	Group LH-mid (n=213)	*p*-value
Female age	31.30 ± 3.08	31.86 ± 2.43	0.169
Male age	35.64 ± 5.65	35.96 ± 5.38	0.696
BMI	24.94 ± 4.25	23.83 ± 3.70	0.062
FSH dose (IU)	2196.01 ± 842.81	2406.95 ± 661.68	0.061
LH dose (IU)	1101.79 ± 782.66	782.66 ± 452.78	0.001
Estradiol level (pg/mL)	2229.94 ± 2107.54	2716.37 ± 1942.41	0.308
Follicles (n)	16.89 ± 12.60	19.69 ± 11.69	0.089
Retrieved oocytes (n)	12.66 ± 10.54	14.77 ± 9.81	0.126
Oocyte yield (%)	72.67 ± 24.42	73.73 ± 20.03	0.716
MII oocyte rate (%)	71.62 ± 20.14	70.41 ± 19.81	0.664
Fertilization rate (%)	79.45 ± 20.04	77.37 ± 21.53	0.506
Normal cleavage speed rate (%)	76.71 ± 23.63	75.40 ± 23.52	0.709
D3 high -quality embryos rate (%)	46.67 ± 24.57	40.36 ± 25.43	0.266
Blastocyst development rate (%)	46.76 ± 33.44	47.67 ± 32.68	0.887
Frozen embryos (n)	5.83 ± 5.21	5.69 ± 4.61	0.877
Endometrial thickness (mm)	10.76 ± 2.54	10.54 ± 2.03	0.635
Embryos transferred (n)	1.27 ± 0.50	1.22 ± 0.60	0.845
Cycles with embryo transfer (%)	61.43 (43/70)	50.23 (107/213)	0.129
Implantation rate (%)	41.5 ± 46.2	41.0 ± 46.0	0.951
Pregnancy rate (%)	48.84 (21/43)	47.66 (51/107)	0.896
Miscarriage rate (%)	9.52 (2/21)	6.94 (3/51)	0.625

Note: values are mean ± standard deviation, unless otherwise noted. ICSI - intracytoplasmic sperm injection, COS - controlled ovarian stimulation, rFSH - recombinant follicle stimulating hormone, rLH - recombinant luteinizing hormone, BMI - body mass index, IU - international unit, MII - metaphase II, D3 - day 3 of embryo development.

In patients aged ≥ 35 years, higher blastocyst development rate was observed in Group LH-mid compared to Group LH-start (38.5 ± 32.3 vs. 28.8 ± 30.4, *p*=0.006) (Table 3).

**Table 3:** Descriptive analysis of patients’ demographics, response to COS and laboratorial ICSI outcomes in patients aged ≥ 35 years

Variable	Group LH-start (n=253)	Group LH-mid (n=742)	*p*-value
Female age	39.17 ± 2.92	39.09 ± 3.0	0.171
Male age	40.84 ± 6.39	40.58 ± 6.20	0.597
BMI	24.63 ± 3.84	24.61 ± 4.07	0.945
FSH dose (IU)	2550.95 ± 790.25	2613.30 ± 652.36	0.260
LH dose (IU)	1272.49 ± 398.39	1081.56 ± 468.49	0.005
Estradiol level (pg/mL)	1960.59 ± 1957.56	1765.36 ± 1813.44	0.361
Follicles (n)	10.09 ± 8.11	10.64 ± 7.99	0.346
Retrieved oocytes (n)	7.11 ± 5.94	7.71 ± 6.39	0.193
Oocyte yield (%)	69.411 ± 24.39	69.990 ± 25.094	0.750
MII oocyte rate (%)	73.86 ± 23.66	72.71 ± 24.88	0.531
Fertilization rate (%)	76.22 ± 24.16	72.85 ± 26.72	0.102
Normal cleavage speed rate (%)	73.36 ± 30.53	72.47 ± 30.21	0.713
D3 high -quality embryos rate (%)	42.50 ± 26.65	40.63 ± 24.29	0.633
Blastocyst development rate (%)	28.77 ± 30.39	38.46 ± 32.32	0.006
Frozen embryos (n)	3.83 ± 4.59	3.46 ± 3.50	0.496
Endometrial thickness (mm)	10.44 ± 2.13	10.61 ± 2.50	0.490
Embryos transferred (n)	1.35 ± 0.62	1.28 ± 0.52	0.211
Cycles with embryo transfer (%)	54.54 (138/253)	55.06 (416/742)	0.714
Implantation rate (%)	18.8 ± 37.2	22.3 ± 38.6	0.361
Pregnancy rate (%)	22.46 (31/138)	27.40 (114/416)	0.266
Miscarriage rate (%)	12.90 (4/31)	16.67 (19/114)	0.784

Note: values are mean ± standard deviation, unless otherwise noted. ICSI - intracytoplasmic sperm injection, COS - controlled ovarian stimulation, rFSH - recombinant follicle stimulating hormone, rLH - recombinant luteinizing hormone, BMI - body mass index, IU - international unit, MII - metaphase II, D3 - day 3 of embryo development.

In patients with low response to COS (< 5 retrieved oocytes), there were significant differences between the LH-start and the LH-mid groups regarding the female age (38.4 ± 4.3 vs. 39.5 ± 3.8, *p*=0.011, respectively) and body mass index (24.1 ± 3.1 vs. 24.9 ± 4.5, *p*=0.033, respectively), therefore, the analysis was adjusted for these variables. Significantly higher fertilization rate (68.3 ± 2.5 vs. 78.6 ± 3.7, *p*=0.023), blastocyst development rate (22.5 ± 7.2 vs. 44.7 ± 6.2, *p*=0.022) and implantation rate (13.7 ± 37.0 vs. 18.2 ± 37.8, *p*<0.001) were observed in Group LH-mid (Table 4).

**Table 4:** Descriptive analysis of patients’ demographics, response to COS and laboratorial ICSI outcomes in patients with poor response to COS (≤ 4 retrieved oocytes)

Variable	Group LH-start (n=122)	Group LH-mid (n=299)	*p*-value
Female age	38.39 ± 4.344	39.48 ± 3.806	0.011
Male age	39.70 ± 6.48	39.30 ± 6.89	0.583
BMI	24.10 ± 3.10	24.96 ± 4.47	0.033
FSH dose (IU)	2581.20 ± 73.19	2705.05 ± 46.55	0.155
LH dose (IU)	1236.02 ± 34.21	1062.35 ± 54.33	0.007
Estradiol level (pg/mL)	715.07 ± 136.14	768.29 ± 80.55	0.737
Follicles (n)	4.24 ± 0.23	4.45 ± 0.145	0.435
Retrieved oocytes (n)	2.32 ± 0.12	2.39 ± 0.08	0.631
Oocyte yield (%)	57.97 ± 3.00	56.51 ± 1.92	0.685
MII oocyte rate (%)	71.02 ± 2.12	77.82 ± 3.29	0.084
Fertilization rate (%)	68.35 ± 2.50	78.56 ± 3.69	0.023
Normal cleavage speed rate (%)	26.08 ± 4.06	30.93 ± 2.88	0.333
D3 high -quality embryos rate (%)	39.48 ± 7.98	64.79 ± 11.45	0.075
Blastocyst development rate (%)	22.51 ± 7.18	44.66 ± 6.24	0.022
Frozen embryos (n)	1.20 ± 0.18	1.43 ± 0.15	0.327
Endometrial thickness (mm)	9.90 ± 0.34	9.89 ± 0.19	0.985
Embryos transferred (n)	1.13 ± 0.05	1.13 ± 0.03	0.971
Cycles with embryo transfer (%)	43.44 (53/122)	41.86 (126/301)	0.760
Implantation rate (%)	13.7 ± 37.0	18.2 ± 37.8	<0.001
Pregnancy rate (%)	13.21 (7/53)	19.05 (24/126)	0.354
Miscarriage rate (%)	0.00 (0.0)	4.17 (1/24)	0.723

Note: values are mean ± standard deviation, unless otherwise noted. ICSI - intracytoplasmic sperm injection, COS - controlled ovarian stimulation, rFSH - recombinant follicle stimulating hormone, rLH - recombinant luteinizing hormone, BMI - body mass index, IU - international unit, MII - metaphase II, D3 - day 3 of embryo development.

In patients with normal response to COS (> 4 retrieved oocytes), no significant differences were observed between the groups (Table 5).

**Table 5:** Descriptive analysis of patients’ demographics, response to COS and laboratorial ICSI outcomes in patients with normal response to COS (> 5 retrieved oocytes).

Variable	Group LH-start (n=201)	Group LH-mid (n=656)	*p*-value
Female age	36.90 ± 4.32	36.55 ± 3.99	0.297
Male age	39.68 ± 6.67	38.76 ± 5.91	0.085
BMI	24.06 ± 4.32	24.20 ± 3.75	0.656
FSH dose (IU)	2453.75 ± 814.53	2525.69 ± 624.40	0.186
LH dose (IU)	1258.55 ± 403.92	925.81 ± 414.41	< 0.001
Estradiol level (pg/mL)	2994.27 ± 2084.32	2480.91 ± 1937.77	0.055
Follicles (n)	15.92 ± 9.84	16.41 ± 9.46	0.518
Retrieved oocytes (n)	11.89 ± 7.50	12.44 ± 7.58	0.362
Oocyte yield (%)	77.21 ± 15.90	77.13 ± 17.00	0.950
MII oocyte rate (%)	71.87 ± 17.27	72.34 ± 18.92	0.752
Fertilization rate (%)	76.42 ± 19.11	75.81 ± 19.96	0.714
Normal cleavage speed rate (%)	25.67 ± 24.79	25.37 ± 25.27	0.889
D3 high -quality embryos rate (%)	41.24 ± 23.54	40.47 ± 23.85	0.813
Blastocyst development rate (%)	34.76 ± 30.91	40.64 ± 31.37	0.073
Frozen embryos (n)	5.70 ± 5.08	4.76 ± 4.13	0.124
Endometrial thickness (mm)	10.77 ± 2.33	10.81 ± 2.40	0.865
Embryos transferred (n)	1.41 ± 0.65	1.39 ± 0.58	0.784
Cycles with embryo transfer (%)	61.69 (124/201)	59.79 (391/654)	0.680
Implantation rate (%)	27.7 ± 41.7	28.6 ± 41.6	0.824
Pregnancy rate (%)	33.87 (42/124)	35.29 (138/391)	0.829
Miscarriage rate (%)	14.29 (6/42)	13.04 (18/138)	0.800

Note: values are mean ± standard deviation, unless otherwise noted. ICSI - intracytoplasmic sperm injection, COS - controlled ovarian stimulation, rFSH - recombinant follicle stimulating hormone, rLH - recombinant luteinizing hormone, BMI - body mass index, IU - international unit, MII - metaphase II, D3 - day 3 of embryo development.

## Discussion

In this study, we aimed at investigating whether the timing of rLH addition to rFSH during COS impacts ovarian response and the outcomes of ICSI cycles in different female populations. We observed that patients with low response to COS benefited from the addition of LH in the mid-follicular phase, which resulted in improved fertilization, blastocyst development and implantation rates compared to those in which LH was started concomitantly with FSH administration.

Our findings are in line with a previous study that demonstrated the value of LH supplementation in the mid-follicular phase in POR patients (Gizzo *et al*., 2015), by rescuing the ongoing cycle through the compensation of an initial slow ovarian response (Alviggi *et al*., 2018). The use of LH supplementation creates a more physiological gonadotrophic surrounding and balances the severe deprivation of endogenous LH in GnRH antagonist cycles. Nonetheless, the excessive production of LH, derived from the concomitant administration of exogenous FSH and LH from the beginning of cycle, may cause follicle atresia by exceeding the window of LH concentration for the normal follicular function (Filicori *et al*., 2003; Palermo, 2007). In addition, growing follicles become progressively sensitive to and ultimately dependent on LH activity for their development, a fact that may justify its supplementation in the mid to late follicular phase (Shoham, 2002).

Even though we have failed to demonstrate significant differences between the LH timing groups in terms of number of retrieved oocytes and mature oocytes, we observed significant differences in the rates of fertilization and blastulation, which suggests that the oocytes were of higher quality, which in turn lead to a higher implantation rate in the Group LH-mid compared to Group LH-start. Despite patients treated with LH, regardless timing of start, possess reduced apoptotic cumulus cells (CC) (Ruvolo *et al*., 2007), those in which LH was administered later in the follicular phase showed higher concentrations of CC RNA expression and intra-follicular growth factors (Barberi *et al*., 2012). Another explanation for the improved implantation rate besides higher blastocyst implantation potential might be related to the influence of LH on the upregulation of endometrial LH receptors, thus enhancing endometrial receptivity (Tesarik *et al*., 2003).

Previous studies have showed the beneficial effects of LH supplementation in POR patients. Lehert *et al*. (2014) found significantly higher number of retrieved oocytes and an increase of 30% in clinical pregnancy rates in a meta-analysis that included 40 randomized controlled trials (RCTs). A recent study showed a half-fold change in the incidence of total pregnancy outcome failure (defined as a combination of biochemical pregnancy, early spontaneous miscarriage, late spontaneous miscarriage, and ectopic pregnancy) in LH-supplemented cycles compared to r-FSH monotherapy (Humaidan *et al*., 2017). It is important to highlight that the definition of POR may have biased the outcomes of the studies. For example, a systematic review of 47 RCTs studies found 41 different descriptions for POR and each definition was used by no more than three RCTs (Polyzos & Devroey, 2011). Nonetheless, another study showed that nearly 80% of the IVF clinics participating in a worldwide survey used ‘number of follicles produced’ to interpret POR (Patrizio *et al*., 2015).

The present study adds to general knowledge of POR management; however, it was not without limitations. The limitations included the retrospective design and limited sample size in subpopulations. In addition, the reduced clinical outcomes related to POR patients may hamper the true estimation of the differences between the stimulation groups in terms of pregnancy and miscarriage rates.

In conclusion, the findings of the present study are clinically relevant and, interpreted along with previous evidence, support the use of LH supplementation in POR patients, which may improve laboratorial and clinical outcomes when started in the mid-follicular phase, in GnRH antagonist ICSI cycles.

## References

[r1] Alviggi C, Clarizia R, Mollo A, Ranieri A, De Placido G (2011). Who needs LH in ovarian stimulation?. Reprod Biomed Online.

[r2] Alviggi C, Humaidan P, Ezcurra D. (2012). Hormonal, functional and genetic biomarkers in controlled ovarian
stimulation: tools for matching patients and protocols. Reprod Biol Endocrinol.

[r3] Alviggi C, Conforti A, Esteves SC, Andersen CY, Bosch E, Bühler K, Ferraretti AP, De Placido G, Mollo A, Fischer R, Humaidan P. (2018). Recombinant luteinizing hormone supplementation in assisted
reproductive technology: a systematic review. Fertil Steril.

[r4] Barberi M, Ermini B, Morelli MB, Ermini M, Cecconi S, Canipari R. (2012). Follicular fluid hormonal profile and cumulus cell gene
expression in controlled ovarian hyperstimulation with recombinant FSH:
effects of recombinant LH administration. J Assist Reprod Genet.

[r5] Bosch E, Labarta E, Crespo J, Simón C, Remohí J, Pellicer A. (2011). Impact of luteinizing hormone administration on
gonadotropin-releasing hormone antagonist cycles: an age-adjusted
analysis. Fertil Steril.

[r6] Filicori M, Cognigni GE, Ciampaglia W. (2003). What clinical evidence for an LH ceiling?. Hum Reprod.

[r7] Gizzo S, Andrisani A, Noventa M, Manfè S, Oliva A, Gangemi M, Nardelli GB, Ambrosini G. (2015). Recombinant LH supplementation during IVF cycles with a
GnRH-antagonist in estimated poor responders: A cross-matched pilot
investigation of the optimal daily dose and timing. Mol Med Rep.

[r8] Hill MJ, Levens ED, Levy G, Ryan ME, Csokmay JM, DeCherney AH, Whitcomb BW (2012a). The use of recombinant luteinizing hormone in patients undergoing
assisted reproductive techniques with advanced reproductive age: a
systematic review and meta-analysis. Fertil Steril.

[r9] Hill MJ, Levy G, Levens ED (2012b). Does exogenous LH in ovarian stimulation improve assisted
reproduction success? An appraisal of the literature. Reprod Biomed Online.

[r10] Humaidan P, Bungum M, Bungum L, Yding Andersen C (2004). Effects of recombinant LH supplementation in women undergoing
assisted reproduction with GnRH agonist down-regulation and stimulation with
recombinant FSH: an opening study. Reprod Biomed Online.

[r11] Humaidan P, Chin W, Rogoff D, D’Hooghe T, Longobardi S, Hubbard J, Schertz J (2017). Efficacy and safety of follitropin alfa/lutropin alfa in ART: a
randomized controlled trial in poor ovarian responders. Hum Reprod.

[r12] Lehert P, Kolibianakis EM, Venetis CA, Schertz J, Saunders H, Arriagada P, Copt S, Tarlatzis B (2014). Recombinant human follicle-stimulating hormone (r-hFSH) plus
recombinant luteinizing hormone versus r-hFSH alone for ovarian stimulation
during assisted reproductive technology: systematic review and
meta-analysis. Reprod Biol Endocrinol.

[r13] Matorras R, Prieto B, Exposito A, Mendoza R, Crisol L, Herranz P, Burgués S (2011). Mid-follicular LH supplementation in women aged 35-39 years
undergoing ICSI cycles: a randomized controlled study. Reprod Biomed Online.

[r14] Mochtar MH, Danhof NA, Ayeleke RO, Van der Veen F, van Wely M (2017). Recombinant luteinizing hormone (rLH) and recombinant follicle
stimulating hormone (rFSH) for ovarian stimulation in IVF/ICSI
cycles. Cochrane Database Syst Rev.

[r15] Mochtar MH, Van der V, Ziech M, van Wely M (2007). Recombinant Luteinizing Hormone (rLH) for controlled ovarian
hyperstimulation in assisted reproductive cycles. Cochrane Database Syst Rev.

[r16] Palermo G, Joris H, Devroey P, Van Steirteghem AC (1992). Pregnancies after intracytoplasmic injection of single
spermatozoon into an oocyte. Lancet.

[r17] Palermo R. (2007). Differential actions of FSH and LH during
folliculogenesis. Reprod Biomed Online.

[r18] Patrizio P, Vaiarelli A, Levi Setti PE, Tobler KJ, Shoham G, Leong M, Shoham Z. (2015). How to define, diagnose and treat poor responders? Responses from
a worldwide survey of IVF clinics. Reprod Biomed Online.

[r19] Polyzos NP, Devroey P (2011). A systematic review of randomized trials for the treatment of
poor ovarian responders: is there any light at the end of the
tunnel?. Fertil Steril.

[r20] Raju GAR, Chavan R, Deenadayal M, Gunasheela D, Gutgutia R, Haripriya G, Govindarajan M, Patel NH, Patki AS (2013). Luteinizing hormone and follicle stimulating hormone synergy: A
review of role in controlled ovarian hyper-stimulation. J Hum Reprod Sci.

[r21] Ruvolo G, Bosco L, Pane A, Morici G, Cittadini E, Roccheri MC (2007). Lower apoptosis rate in human cumulus cells after administration
of recombinant luteinizing hormone to women undergoing ovarian stimulation
for in vitro fertilization procedures. Fertil Steril.

[r22] Shoham Z. (2002). The clinical therapeutic window for luteinizing hormone in
controlled ovarian stimulation. Fertil Steril.

[r23] Tesarik J, Hazout A, Mendoza C. (2003). Luteinizing hormone affects uterine receptivity independently of
ovarian function. Reprod Biomed Online.

[r24] Wong PC, Qiao J, Ho C, Ramaraju GA, Wiweko B, Takehara Y, Nadkarni PV, Cheng LC, Chen HF, Suwajanakorn S, Vuong TN. (2011). Current opinion on use of luteinizing hormone supplementation in
assisted reproduction therapy: an Asian perspective. Reprod Biomed Online.

